# Predicting glioblastoma prognosis networks using weighted gene co-expression network analysis on TCGA data

**DOI:** 10.1186/1471-2105-13-S2-S12

**Published:** 2012-03-13

**Authors:** Yang Xiang, Cun-Quan Zhang, Kun Huang

**Affiliations:** 1 Department of Biomedical Informatics, The Ohio State University, Columbus, USA; 2 Department of Mathematics, West Virginia University, Morgantown, USA; 3 The Comprehensive Cancer Center Biomedical Informatics Shared Resource, The Ohio State University, Columbus, USA

## Abstract

**Background:**

Using gene co-expression analysis, researchers were able to predict clusters of genes with consistent functions that are relevant to cancer development and prognosis. We applied a weighted gene co-expression network (WGCN) analysis algorithm on glioblastoma multiforme (GBM) data obtained from the TCGA project and predicted a set of gene co-expression networks which are related to GBM prognosis.

**Methods:**

We modified the Quasi-Clique Merger algorithm (QCM algorithm) into edge-covering Quasi-Clique Merger algorithm (eQCM) for mining weighted sub-network in WGCN. Each sub-network is considered a set of features to separate patients into two groups using K-means algorithm. Survival times of the two groups are compared using log-rank test and Kaplan-Meier curves. Simulations using random sets of genes are carried out to determine the thresholds for log-rank test p-values for network selection. Sub-networks with p-values less than their corresponding thresholds were further merged into clusters based on overlap ratios (>50%). The functions for each cluster are analyzed using gene ontology enrichment analysis.

**Results:**

Using the eQCM algorithm, we identified 8,124 sub-networks in the WGCN, out of which 170 sub-networks show p-values less than their corresponding thresholds. They were then merged into 16 clusters.

**Conclusions:**

We identified 16 gene clusters associated with GBM prognosis using the eQCM algorithm. Our results not only confirmed previous findings including the importance of cell cycle and immune response in GBM, but also suggested important epigenetic events in GBM development and prognosis.

## Background

The rapid development of high throughput gene expression profiling technology such as microarray and high throughput sequencing has enabled the development of many new bioinformatics data analysis methods for identifying disease related genes, characterizing disease subtypes and discovering gene signatures for disease prognosis and treatment prediction. For instance, in breast cancer research, a supervised approach was adopted to select 70 genes as biomarkers for breast cancer prognosis [1,2] and was successfully tested in clinical settings [3]. However, a major drawback of such approach is that the selected gene features are usually not functionally related and hence cannot reveal key biological mechanisms and processes behind the difference of the two patient groups.

In order to overcome this issue and identify functionally related genes associated with disease development and prognosis, several approaches have been adopted. One of such approaches is to use gene co-expression analysis. For instance, in [4] and [5] , we carried out gene co-expression network analysis for biomarker discovery in different types of cancers.

The goal of gene co-expression network (GCN) analysis is to identify group of genes which are highly correlated in expression levels across multiple samples. The genes in the same co-expression sub-network are often enriched with similar functions. The metric to measure the correlation is usually the correlation coefficient (e.g., Pearson correlation coefficient or PCC) between expression profiles of two genes [6-8]. Then for each dataset, a weighted graph can be derived with the vertices being the genes and the weights of the edges being the PCC values between the two gene expression profiles. However, many network mining algorithms take only binary edges by imposing a threshold on the PCC values (i.e., two genes are connected by an edge only if the PCC value between them is higher than a pre-defined threshold) and transforming the network into a sparse unweighted gene co-expression network (UWGCN). For instance, in [6] , an algorithm called CODENSE was developed to identify frequent UWGCNs from multiple datasets and this method has been applied to cancer biomarker discovery. Issues with the UWGCN approach include how to determine the threshold of PCC values and the threshold may be too rigid to include edges with weights around that threshold. Thus weighted GCN (WGCN) methods have been developed.

For WGCN, Stephen Horvath's group has developed a series of methods for identifying gene clusters which are highly correlated using hierarchical clustering based approach [7,9,10]. This method was applied to identify disease associated genes such as the ASPM gene in glioblastoma [9]. However, there are several drawbacks of using the hierarchical clustering approach. First, it does not allow direct control over the intracluster connectivity such that the vertices within a cluster have high correlations on average. Second, the clustering approach does not allow shared genes between two sub-networks even though in biology, many genes have multiple functions and can be shared by multiple functional groups and dense sub-networks. Finally, clusters identified using this approach are often large (e.g., more than 100 genes), thus smaller gene networks which contain subtle functional information may not be detected.

In this paper, we take advantage of the dense sub-network finding method in the graph mining community and apply it to mine functional networks using the WGCN approach to identify dense co-expression sub-networks in glioblastoma. Specifically, using The Cancer Genome Atlas (TCGA) data sets, we identified *co-expressed sub-networks *(*sub-networks *for short in the following) for genes then we tested if these sub-networks can be used as features to separate patients into groups with different survival times. Using this approach, we identified 16 gene networks associated with GBM prognosis. Our results not only confirmed previous findings in GBM, but also suggested important epigenetic events (histone acetylation) in GBM development and prognosis.

## Methods

### Gene expression dataset for GBM

We downloaded gene expression data from the Cancer Genome Atlas (TCGA) project webpage (http://cancergenome.nih.gov, downloaded on 11/24/2010) for all GBM patients with gene expression data generated using Affymetrix HU133 Genechip. We also downloaded all available public clinical data including survival information. In total, we selected 361 patients with complete data (i.e., each has one set of gene expressions, one set of microRNA expressions, and public clinical information). Among them, 345 have a valid vital status (i.e., either LIVING or DECEASED) and they are good for survival tests. The gene expression data were normalized using RMA normalization as described in the TCGA NCI Wiki.

### Building WGCN for genes

After normalization a total of 12,042 unique genes were available. PCC were computed between every pair of genes. We then set the genes to be the vertices of the WGCN with the absolute values of PCC (|PCC|) being weights of the edges.

### Identify quasi-cliques in the WGCNs

We first define the density of a weighted network with *N *vertices with ***w_ij _***being the weight, normalized between 0 and 1, between vertices ***v_i _***and ***v_j _***(*i = 1, 2,..., N, j = 1, 2,..., N*, and *i≠j *) as $d=\frac{\sum_{i=1}^{N-1}\sum_{j=i+1}^{N}w_{ij}}{N\left(N-1\right)/2}$

For mining densely connected networks in the WGCN, our approach is based on an existing algorithm previously developed for mining weighted networks [11]. Different from many graph mining approaches (e.g., [12]) that focus on unweighted graphs, the algorithm of [11] targets primarily at identifying dense components (or sub-networks) in a weighted graph (i.e., each edge has a weight), although it is called Quasi-Clique Merger (QCM). To mine dense-sub-networks in a gene-coexpression network, we slightly revise the original QCM algorithm by removing the hierarchical construction which does not contribute to our dense-sub-network finding, and changing the new search start condition from checking vertex coverage to checking edge coverage to ensure that each edge with its weight no less than the weight threshold (γ times the maximum edge weight) will be covered by at least one dense-sub-network. The revised algorithm is sketched below:

**Algorithm 1 eQCM **(**edge-covering Quasi-Clique Merger, a revised version of QCM **[11]. Input G=(V, E), γ, λ, t, β, Output: *C *)

1: Sort edges in E in descending order of their weights;

2: **for **i = 1:μ {e_μ _is the last edge in the above sorted list with weight ≥γ·e_1_}

3: **if **e_i _is an edge in any sub-network in *C*

4: **continue**;

5: **endif**

6: C = V(e_i_); U = V \ V(e_i_);

7: **while **max_{v∈U}_(*contribute*(v,C)) ≥ *threshold*

8: C = C ∪ {v};

9: U = U \ {v};

10: **endwhile**

11: *C *= *C *∪ {C};

12: **endfor**

13: Merging highly overlapped sub-networks in *C *with respect to β;

14: Output *C *;

To be consistent with the original QCM algorithm [11], *contribute *(v, C) is defined as the ratio of the edge weight increase of G(C) on adding the vertex v, over the size of C, and *threshold *is $\left(1-\frac{1}{2\lambda \left(|C|+t\right)}\right)$ *density (G(C)), which is determined by the input parameter λ, t, the size of C, and the density of the sub-network induced by C. Readers may refer to [11] for additional details. The last second step (merging) is the step 4 in the original QCM algorithm. Since we are interested in identifying gene sub-networks with potential consistent functions, we select only the sub-networks with at least 10 genes to facilitate gene function enrichment analysis.

### Survival test for identified networks

For each sub-network, we test if the genes in it can be used as potential prognostic markers for predicting GBM survival. For a network with *k *genes, we extract the expression values for them for all patients and use them as the feature vectors for patients. The patients are then divided into two groups using the unsupervised K-means clustering algorithm (K = 2, 100 time replicates, correlation distance measure).

The survival times for the two patient groups are plotted in Kaplan-Meier curves and the difference between the two groups is tested using log-rank test (code at http://www.mathworks.com/matlabcentral/fileexchange/20388). P-values for the log-rank tests for all the identified networks are recorded.

### Select representative sub-networks with significant p-values

Since many of the identified networks have large overlaps, we cannot directly apply multiple test compensation method such as the Bonferroni correction as the tests are not independent and such correction would be too conservative. Instead, we design a randomized test to determine the false discovery ratio (FDR) for selecting significant sub-networks.

For an N-gene sub-network, we randomly selected a list of genes from the entire gene list in the dataset such that the expected length of the selected gene list is N. Then we repeat the survival test process as described above. Such random test is repeated 1000 times. The lower 5^th ^percentile of the 1000 p-values is used as the threshold for p-value for selecting sub-networks with significant prognostic power. Since we have a large number of sub-networks and cannot carry out 1000 random tests for every possible N, we do such random tests for N = 1*10^1^ , 2*10^1^ ,...9*10^1^ , 1*10^2^ , 2*10^2^ ,... and the p-value thresholds are p_10_, p_20_,..., p_100_, p_200_,... Our results show that the p-value thresholds are close when N are close. Thus for a sub-network with size N', its p-value for survival test is compared to *p_i _*where $i=\left\lfloor \frac{N^{\prime }}{10^{\left\lfloor \text{lg}N^{\prime }\right\rfloor }}\right\rfloor *10^{\left\lfloor \text{lg}N,\prime \right\rfloor }$ to determine if it is significant. For example, a gene list with 28 genes compares its p-value to *p_20_*, and a gene list with 250 genes compare its p-value to *p_200_*.

We also noticed that many of the selected significant sub-networks have substantial overlaps and they form exclusive clusters. To identify such clusters, we iteratively merge networks with substantial overlaps (i.e, the overlap ratio *r *between two networks is larger than 50%) into clusters. The overlap ratio between two sub-networks G_1_=(V_1_, E_1_) and G_2_=(V_2_, E_2_) is defined as $\frac{\left|V_{1}\cap V_{2}\right|}{min\left(\left|V_{1}\right|,|V_{2}|\right)}$ Then we pick the sub-network with the lowest p-value in each cluster as the representative sub-network for further analysis.

For the representative sub-networks, we used TOPPGene (http://toppgene.cchmc.org) for gene ontology and pathway enrichment analysis without Bonferroni correction.

## Results

**Figure 1:**
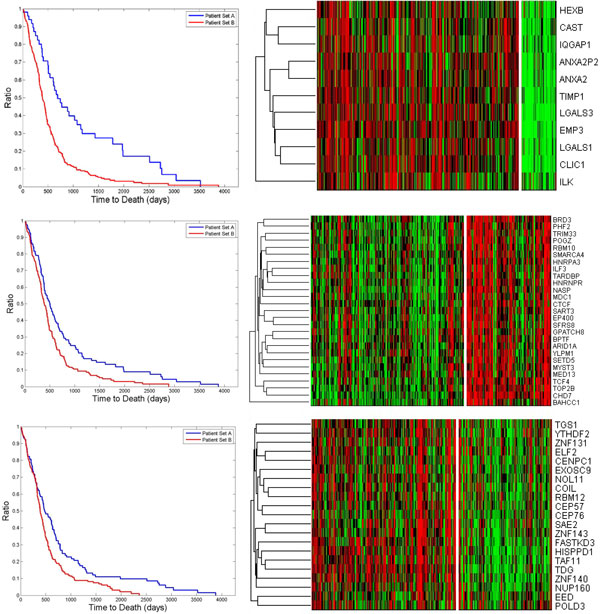
**Kaplan-Meier curves and heatmaps for two groups of patients with significantly different survival times identified using three networks**. **Top**: Left - The Kaplan-Meier curve for the patients separated using the immune response network #1. Right - The heatmap of the gene expression values for the genes in the representative network #1. The vertical white line indicates the separation between short survival (left to the white line) and long survival (right to the white line) groups. The density of the representative network is 0.6928 and the p-value is 5.7 × 10^-5^. **Middle**: The Kaplan-Meier curve and heatmap for the chromatin modification network #6. The density of the representative network is 0.6951 and the p-value is 0.0016. **Bottom**: The Kaplan-Meier curve and heatmap for the mitotic cycle network #13. The density of the representative network is 0.6764 and the p-value is 0.006774.

Using the eQCM algorithm (γ = 0.7, λ = 1, t = 1, β = 0.99999), we identified 8,124 sub-networks with at least ten vertices in the WGCN. The survival tests were then carried out on them and 866 show p-values less than 0.05. In addition, random tests were performed to obtain p_10_, p_20_,..., p_90_, p_100_,..., p_500 _and all of them are smaller than 0.01. 170 sub-networks with significant p-values were selected and their densities range from 0.612 to 0.862. Then sub-networks with substantial overlaps (overlap ratio > 50%) were iteratively merged into sixteen clusters. The representative sub-networks for every cluster and their p-values and enriched GO functions are shown in Table 1. For cluster 1, the representative sub-network is highly enriched with genes involved in extracellular matrix organization (*p *= 8.22 × 10^-7^) which also engage in many important biological processes such as cell-cell signaling and immune responses. Indeed, the entire set of genes in cluster 1 are highly enriched with immune system process genes (*p *= 1.01 × 10^-46^). Figure 1 shows examples of the Kaplan-Meier curves for some of the representative sub-networks in separating the patients using the unsupervised K-means algorithm, and heatmaps for these sub-networks.

**Table 1:** List of representative networks with log-rank test p-values.

Cluster #	# of unique genes	Representative network size	Log-rank test p-value	Top enriched GO terms	Member genes of the representative network
1	**284**	11	5.7 × 10^-5^	**BP**: extracellular matrix organization (8.22 × 10^-7^) **BP **(entire cluster): immune system process (1.01 × 10^-46^) **MF**: enzyme inhibitor activity (2.28 × 10^-7^) **CC**: proteinacious extracellular matrix (7.32 × 10^-7^)	CLIC1, ILK, LGALS1, LGALS3, ANXA2, TIMP1, ANXA2P2, IQGAP1, EMP3, CAST, HEXB

2	**43**	22	1.31 × 10^-4^	**BP**: chromatin organization (1.91 × 10^-4^) **MF**: deoxycytidyltransferase activity (2.28 × 10^-7^) **CC**: nucleoplasm (7.75 × 10^-5^)	C10orf18, TAF5, SIRT1, FMR1, FBXO11, TCERG1, CXorf45, CASP8AP2, ARID4B, JMJD1C, TAF2, ELF2, CENPC1, ZNF131, NUP153, SUZ12, SR140, ATAD2B, HISPPD1, REV1, PMS1, ZCCHC11

3	**34**	16	2.21 × 10^-4^	**BP**: RNA processing (6.60 × 10^-5^) **MF**: poly(A)-specific ribonuclease activity (1.50 × 10^-3^) **CC**: nuclear speck (9.97 × 10^-5^)	USP52, ZCCHC11, FNBP4, CROP, NKTR, SFRS18, RBM6, RBM5, CCNL2, C21orf66, DMTF1, WSB1, CDK5RAP3, ZNF692, LOC440350, LOC339047

4	**27**	25	4.52 × 10^-4^	**BP**: translation (2.06 × 10^-5^), ncRNA metabolic process (4.01 × 10^-4^) **MF**: structural constituent of ribosome (5.92 × 10^-5^) **CC**: ribonucleoprotein complex (2.03 × 10^-7^)	RPP30, UCK2, BUB3, SMNDC1, SAR1A, MRPS16, GLRX3, TIMM23, UTP11L, HCCS, POLR3C, EIF2B3, MRPL9, SNRPD1, TFB2M, SUMO1, FASTKD3, HSPA14, DUSP11, ATPBD1C, MRPS15, MED28, GTF2B, MRPL22, POLE3

5	**15**	15	6.19 × 10^-4^	**BP**: RNA processing (2.18 × 10^-10^), RNA splicing (7.93 × 10^-8^) **MF**: RNA binding (2.00 × 10^-12^) **CC**: heterogeneous nuclear ribonucleoprotein complex (1.22 × 10^-12^)	JARID1B, RBM12, ADNP, CPSF6, HNRPA3, ILF3, CTCF, HNRPD, HNRNPA0, SART3, HNRPDL, SFPQ, HNRNPR, TARDBP, TLK2

6	**35**	27	0.0016	**BP**: chromatin modification (1.64 × 10^-9^), histone acetylation (5.73 × 10^-6^) **MF**: transcription activator activity (1.18 × 10^-5^) **CC**: nucleolus (1.39 × 10^-8^)	BAHCC1, CHD7, PHF2, TOP2B, TCF4, MYST3, SETD5, POGZ, BRD3, MED13, BPTF, GPATCH8, TARDBP, ILF3, HNRNPR, NASP, MDC1, ARID1A, TRIM33, CTCF, HNRPA3, RBM10, YLPM1, SMARCA4, SART3, SFRS8, EP400

7	**12**	12	0.002747	**BP**: pentose-phosphate shunt , oxidative branch (1.94 × 10^-3^) **MF**: 6-phosphoglucono-lactonase activity (1.29 × 10^-3^) **CC**: ribosome (7.42 × 10^-3^)	PGLS, TMED1, CD320, MRPL4, RFXANK, TMEM161A, CLPP, STX10, TMEM147, EIF3G, C19orf56, UBA52

8	**39**	32	0.002782	**BP**: translation (8.30 × 10^-7^) **MF**: structural constituent of ribosome (8.92 × 10^-9^) **CC**: ribosome (1.59 × 10^-9^), mitochondrion (2.47 × 10^-6^)	COMMD3, HSBP1, ZNF32, SUPT4H1, NFU1, LYRM4, RPS3A, RPS7, SNRPG, HAX1, MED28, UXT, MRPL22, FAM96B, UQCRQ, HBXIP, UBL5, MRPS15, NDUFA2, GTF2B, DUSP11, PSMA5, GTF2A2, PSMB4, ATP5F1, MRPL13, ATPBD1C, MRPL46, MRPL11, MRPS7, WDR61, BNIP1

9	**25**	25	0.003697	**BP**: RNA processing (1.06 × 10^-3^), mitotic cell cycle (1.66 × 10^-3^) **MF**: eukaryotic initiation factor 4G binding (1.29 × 10^-3^), RNA binding (4.08 × 10^-3^) **CC**: chromosomal part (2.64 × 10^-3^)	DDX52, PRPSAP2, YWHAQ, ORC4L, MOBKL3, MYNN, CENPQ, C11orf73, MIS12, HMGN4, C14orf104, FASTKD3, SNRPD1, C4orf27, SFRS3, SUMO1, GIN1, FLJ13611, THAP1, ATPBD1C, DUSP11, EIF4E, PIGF, RY1, NIF3L1

10	**14**	14	0.004707	**BP**: nuclear-transcribed mRNA catabolic process (1.82 × 10^-3^) **MF**: RNA binding (3.02 × 10^-3^) **CC**: BRISC complex (2.94 × 10^-3^)	DDX50, DIP2C, KIAA0157, KIAA1128, KIAA1279, LARP5, PAPD1, RAB11FIP2, SHOC2, TNKS2, UPF2, WAC, WDR37, ZMYND11

11	**22**	16	0.005489	**BP**: type I interferon-mediated signalling (4.49 × 10^-14^) pathway, immune system process (1.79 × 10^-11^) **MF**: MHC class I receptor activity (4.94 × 10^-10^) **CC**: MHC class I protein complex (1.80 × 10^-9^)	CASP1, CASP4, PLSCR1, NMI, SP100, SP110, TRIM22, TRIM6-TRIM34, TRIM21, IFI35, PSMB9, PSMB8, HLA-F, HLA-B, HLA-C, HLA-E

12	**12**	12	0.005663	**BP**: - **MF**: sequence-specific DNA binding transcription factor activity (2.43 × 10^-2^) **CC**: -	ZNF134, ZNF180, ZNF211, ZNF222, ZNF223, ZNF228, ZNF230, ZNF304, ZNF419, ZNF45, ZNF606, ZNF8

13	**21**	21	0.006774	**BP**: mitotic cell cycle (6.43 × 10^-5^) **MF**: RNA trimethylguanosine synthase activity (1.13 × 10^-3^) **CC**: nucleoplasm (4.08 × 10^-4^)	EED, POLD3, ELF2, CENPC1, HISPPD1, ZNF131, RBM12, CEP57, NOL11, COIL, NUP160, CEP76, ZNF140, ZNF143, TDG, TAF11, FASTKD3, TGS1, EXOSC9, YTHDF2, SAE2

14	**18**	18	0.006858	**BP**: arginine biosynthetic process via orthithine (9.69 × 10^-4^) **MF**: argininosuccinate lyase activity 9.67 × 10^-4^) **CC**: organelle envelope (4.11 × 10^-3^)	ASL, ZMYM6, RAB32, CD58, MOBKL1B, TRAM1, CD164, RER1, CCDC109B, CLIC1, CASP4, SQRDL, SERPINB1, MR1, CASP1, CAPG, MGAT4A, ANXA4

15	**22**	22	0.007708	**BP**: multicellular organismal movement (4.97 × 10^-4^) **MF**: ATP-dependent helicase activity (2.22 × 10^-4^) **CC**: endopeptidase Clp complex (1.10 × 10^-3^)	SEC24A, TMF1, KIAA0372, CLCC1, DHX29, SLC30A5, VPS54, CHD1, RPS6KB1, HISPPD1, ETAA1, CENPC1, CLPX, C1orf9, ZNF131, KLHL20, REV1, ZC3H7A, DDX46, NUP153, SMCHD1, PPWD1

16	**15**	15	0.008207	**BP**: ribulose-phosphate 3-epimerase activity (8.069 × 10^-4^) **MF**: mRNA 3'-end processing (1.429 × 10^-3^) **CC**: SPOTS complex (1.574 × 10^-3^)	C15orf15, SELT, COMMD10, UBE2A, TMED2, CNOT8, NMD3, MRPL42, BZW1, NUDT21, SPTLC1, DCTN4, YIPF5, RPE, C20orf30

The p-values associated with the GO terms are based on Fisher's exact tests without Bonferroni correction (http://toppgene.cchmc.org).

## Discussion

In this paper, we carried out a co-expression analysis on GBM gene expression data to screen for biological processes involved in patient prognosis. In previous studies, using co-expression analysis based on clustering algorithm, ASPM has been identified as an important target gene in GBM [9]. ASPM is involved in cell cycle and mitosis functions and many networks with ASPM were identified in our study. We also identified a mitosis related sub-network with a significant p-value in our study (sub-network #13 in Table 1). Besides cell cycle networks, immune response networks also prove to be critical in GBM development as shown in sub-networks #1 and #11, which is consistent with the previous report on the importance of immune and inflammation genes in GBM [13]. As shown in Figure 1, genes in sub-network #1 show higher expression levels in the short survival group. Since a characteristic of GBM is its high metastasis occurrence and extracellular and immune genes play important roles in metastasis, the genes in this group may be potential targets for treatment for reducing metastasis. An interesting observation is that two sub-networks (#2 and #6) related to chromatin modification are identified. Particularly in sub-network #6, histone acetylation genes are highly enriched including well known chromatin modification genes such as CTCF [14] and EP400 [15]. The expression levels of these genes show down-regulation in the short survival group which indicates a possibly reduced histone acetylation activity. Histone acetylation is an important epigenetic event [16] and our findings suggest that epigenetics may play an important role in GBM development and prognosis and ChIP-seq experiments targeting histone acetylation changes associated with GBM development may be necessary. These findings are subject to further cross-validation and experimental investigation. Besides genes, our approach can be applied to identify microRNA modules which show strong association with patient survival and the results can also shed light on microRNA transcription regulation.

## Conclusions

In this paper, we introduced eQCM algorithm for mining dense network clusters in weighted graphs and used this approach to identify 16 gene networks associated with GBM prognosis on weighted gene co-expression network. Our results not only confirmed previous findings including the importance of cell cycle and immune response networks in GBM, but also suggested important epigenetic events in GBM development and prognosis.

## Competing interests

The authors declare that they have no competing interests.

## Authors' contributions

YX carried out the development and implementation of eQCM and survival tests. CQZ originally proposed and designed the QCM algorithm. KH led the project including development of the idea, design of all experiments and writing of the manuscript. All authors edited the manuscript.

## References

[B1] van 't Veer LJ Dai H van de Vijver MJ He YD Hart AA Mao M Peterse HL van der Kooy K Marton MJ Witteveen AT Gene expression profiling predicts clinical outcome of breast cancer Nature 2002 415 6871 530 536 10.1038/415530a 11823860

[B2] van de Vijver MJ He YD van't Veer LJ Dai H Hart AA Voskuil DW Schreiber GJ Peterse JL Roberts C Marton MJ A gene-expression signature as a predictor of survival in breast cancer The New England journal of medicine 2002 347 25 1999 2009 10.1056/NEJMoa021967 12490681

[B3] Buyse M Loi S van't Veer L Viale G Delorenzi M Glas AM d'Assignies MS Bergh J Lidereau R Ellis P Validation and clinical utility of a 70-gene prognostic signature for women with node-negative breast cancer Journal of the National Cancer Institute 2006 98 17 1183 1192 10.1093/jnci/djj329 16954471

[B4] Zhang J Huang K Xiang Y Jin R Using Frequent Co-expression Network to Identify Gene Clusters for Breast Cancer Prognosis Proceedings of the International Joint Conference on Bioinformatics, Systems Biology and Intelligent Computing (IJCBS) 2009 Shanghai: IEEE Computer Society 428 434 10.1109/IJCBS.2009.29 PMC3632312 23615925

[B5] Zhang J Xiang Y Ding L Keen-Circle K Borlawsky TB Ozer HG Jin R Payne P Huang K Using gene co-expression network analysis to predict biomarkers for chronic lymphocytic leukemia BMC bioinformatics 11 Suppl 9 S5 10.1186/1471-2105-11-S9-S5 PMC2967746 21044363

[B6] Hu H Yan X Huang Y Han J Zhou XJ Mining coherent dense subgraphs across massive biological networks for functional discovery Bioinformatics (Oxford, England) 2005 21 Suppl 1 i213 221 10.1093/bioinformatics/bti1049 15961460

[B7] Zhang B Horvath S A general framework for weighted gene co-expression network analysis Statistical applications in genetics and molecular biology 2005 4 Article17 10.2202/1544-6115.1128 16646834

[B8] Pujana MA Han JD Starita LM Stevens KN Tewari M Ahn JS Rennert G Moreno V Kirchhoff T Gold B Network modeling links breast cancer susceptibility and centrosome dysfunction Nature genetics 2007 39 11 1338 1349 10.1038/ng.2007.2 17922014

[B9] Horvath S Zhang B Carlson M Lu KV Zhu S Felciano RM Laurance MF Zhao W Qi S Chen Z Analysis of oncogenic signaling networks in glioblastoma identifies ASPM as a molecular target Proceedings of the National Academy of Sciences of the United States of America 2006 103 46 17402 17407 10.1073/pnas.0608396103 17090670 PMC1635024

[B10] Langfelder P Horvath S WGCNA: an R package for weighted correlation network analysis BMC bioinformatics 2008 9 559 10.1186/1471-2105-9-559 19114008 PMC2631488

[B11] Ou Y Zhang C-Q A new multimembership clustering method Jounral of Industrial and Management Optimization 2007 3 4 619 624

[B12] Newman M Girvan M Finding and evaluating community structure in networks Physical Review E 2004 69 2 026113 10.1103/PhysRevE.69.026113 14995526

[B13] Schwartzbaum JA Huang K Lawler S Ding B Yu J Chiocca EA Allergy and inflammatory transcriptome is predominantly negatively correlated with CD133 expression in glioblastoma Neuro-oncology 12 4 320 327 10.1093/neuonc/nop035 PMC2940608 20308310

[B14] Phillips JE Corces VG CTCF: master weaver of the genome Cell 2009 137 7 1194 1211 10.1016/j.cell.2009.06.001 19563753 PMC3040116

[B15] Fuchs M Gerber J Drapkin R Sif S Ikura T Ogryzko V Lane WS Nakatani Y Livingston DM The p400 complex is an essential E1A transformation target Cell 2001 106 3 297 307 10.1016/S0092-8674(01)00450-0 11509179

[B16] Eberharter A Becker PB Histone acetylation: a switch between repressive and permissive chromatin. Second in review series on chromatin dynamics EMBO reports 2002 3 3 224 229 10.1093/embo-reports/kvf053 11882541 PMC1084017

